# Metastasis of uterine endometrioid carcinoma mimicking upper tract urothelial carcinoma

**DOI:** 10.1016/j.eucr.2025.103195

**Published:** 2025-09-01

**Authors:** Yuji Fujizuka, Shugo Harashima, Yoshiyuki Miyazawa, Seiji Arai, Nozomi Nakajima, Mai Onose, Sho Watanuki, Takanori Shimizu, Yoshitaka Sekine, Hidekazu Koike, Hayato Ikota, Kazuhiro Suzuki

**Affiliations:** a Department of Urology, Gunma University Hospital, 3-39-22, Showa-machi, Maebashi, Gunma, 3718511, Japan; b Clinical Department of Pathology, Gunma University Hospital, 3-39-22, Showa-machi, Maebashi, Gunma, 3718511, Japan

**Keywords:** Diagnostic challenge, Uterine endometrioid carcinoma, Metastasis, Upper-tract urothelial carcinoma, Ureteral cancer

## Abstract

Accurate diagnosis of upper-tract urothelial carcinoma is difficult when urine cytology and ureteroscopy are inconclusive. A 59-year-old woman had undergone total hysterectomy for uterine endometrioid carcinoma 5 years earlier. Follow-up computed tomography demonstrated left hydronephrosis and an enhancing mass in the ureter. Urine cytology was negative, but ureteroscopic biopsies revealed malignant cells that were potentially urothelial carcinoma. The patient received left nephroureterectomy, but histopathology unexpectedly showed metastatic uterine endometrioid carcinoma. This case highlights that metastatic relapse of uterine carcinoma can mimic ureteral cancer on imaging. When cytology and biopsy are inconclusive, prior malignancies should remain in the differential diagnosis.

## Highlights

• Upper tract urothelial carcinoma may be difficult to diagnose before definitive surgery.
• Metastasis of another primary cancer can mimic upper tract urothelial carcinoma.
• Urologists should consider this possibility in a patient with a history of other malignancies.

## 1 Introduction

Upper tract urothelial carcinoma (UTUC) occurs in the renal pelvis and ureter. UTUC is significantly less common than bladder cancer,^1^ but is often invasive at presentation and may already involve regional lymph node metastasis.^2^^,^^3^ UTUC can be diagnosed clinically using imaging techniques, including computed tomography (CT), CT urography (CTU), retrograde urography, cytology, and ureteroscopy.^4^^,^^5^ However, benign strictures or metastases from other primary cancers can mimic UTUC, making a definitive diagnosis difficult.^6^

This report describes a rare case that was presumed to be UTUC but was confirmed pathologically to be metastatic uterine endometrioid carcinoma.

## 2 Case presentation

**Fig. 1:**
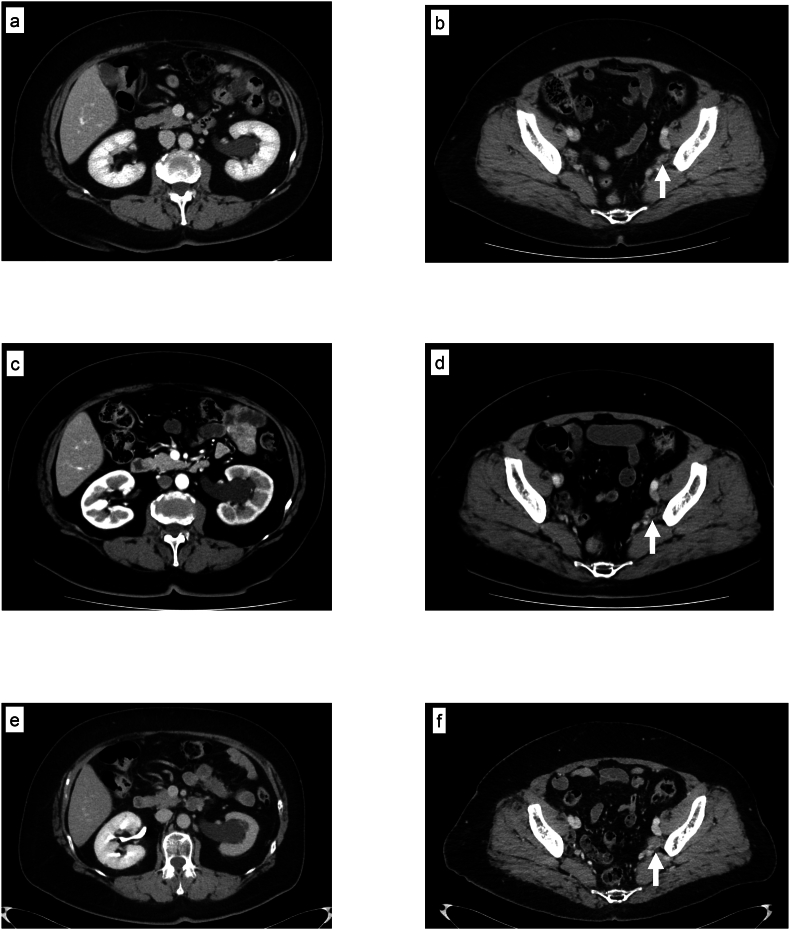
CT images during follow-up. (a, b) Left hydronephrosis (a) and contrast-enhanced lesion in the left distal ureter (b, white arrow) at the time of hydronephrosis detection. (c, d) Slightly decreased enhancement of the left kidney with hydronephrosis (c) and enhanced lesion of same size in the left ureter (d, white arrow) at two months after detection. (e, f) Atrophy and diminished enhancement of the left kidney (e) and an increased size of enhanced lesion (34 × 20 × 20 mm^3^) in the left ureter (f, white arrow) at five months after detection.

A 59-year-old Japanese woman underwent total laparoscopic hysterectomy with bilateral salpingo-oophorectomy and pelvic lymphadenectomy for stage IA (pT1aN0M0) endometrioid carcinoma of the uterus. After the surgery, she received routine surveillance, including tumor marker tests every 3–6 months and annual contrast-enhanced CT. The patient remained disease-free for 5 years, but CT then revealed left hydronephrosis and a slightly enhanced lesion in the distal left ureter, suggesting ureteral cancer (Fig. 1a and b). Serum CA125 levels (7.4 U/mL) were within the normal range, and cystoscopy and urine cytology were negative throughout the follow-up period. She could not undergo magnetic resonance imaging due to claustrophobia and also refused to receive retrograde urography or ureteroscopy. Two months later, CT identified a slightly decreased enhancement of the left kidney and a lesion of the same size in the left ureter (Fig. 1c and d). Five months after left hydronephrosis was detected, CT revealed atrophy and diminished enhancement of the left kidney and increased size of the lesion in the left ureter with no lymph node enlargement, suggesting locally invasive ureteral cancer (clinical T3N0) (Fig. 1e and f).

**Fig. 2:**
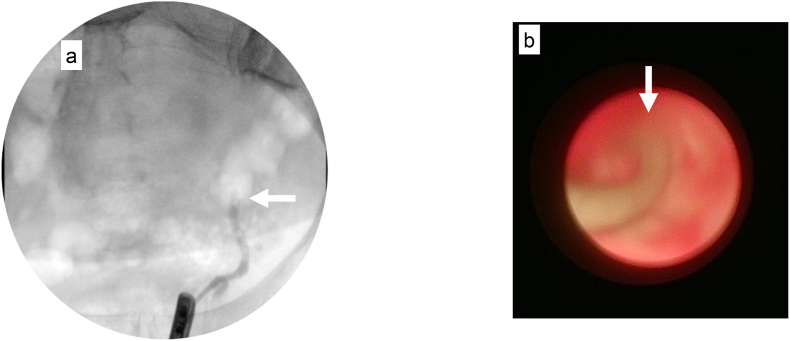
Findings on retrograde urography and ureteroscopy. (a) The lower left ureter showed a filling defect at retrograde urography (white arrow). (b) The guide wire (white arrow) could not pass through the obstructed lesion in the left ureter at ureteroscopy.

**Fig. 3:**
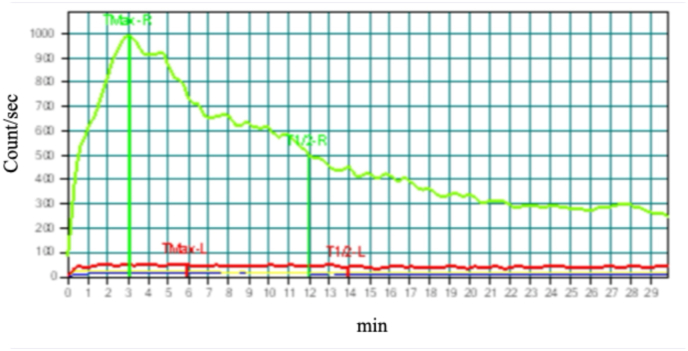
99mTc-MAG3 renogram. MAG3 clearance of the right kidney (green line) and the left kidney (red line) at five months after detection of left hydronephrosis.

The patient finally agreed to undergo further diagnostic tests. Retrograde urography of the left ureter revealed a filling defect (Fig. 2a), and the tumor surface could not be visualized by ureteroscopy due to severe ureteral obstruction at the lesion (Fig. 2b). Ureteroscopic tissue biopsy of the obstructed lesion pathologically identified malignant cells, which were potentially urothelial carcinoma. [^99m^Tc]Tc-mercaptoacetyltriglycine (99mTc-MAG3) renography confirmed normal function of the right kidney (MAG3 clearance of 282.6 ml/min) and a non-functioning left kidney (MAG3 clearance of 24.6 ml/min) (Fig. 3).

**Fig. 4:**
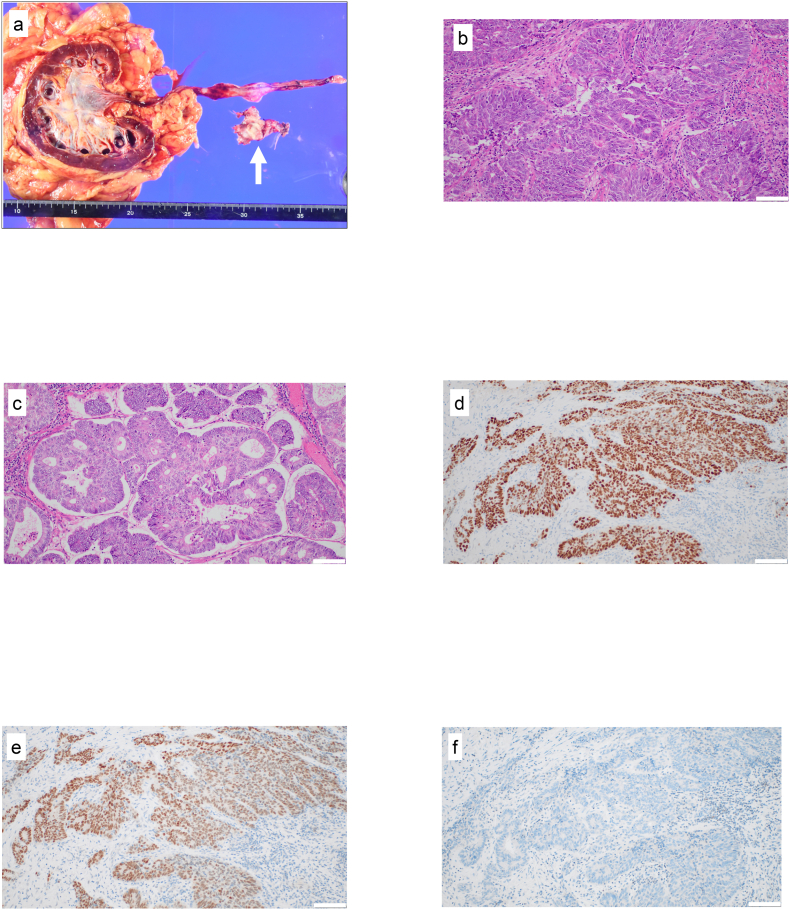
Pathological images of total nephroureterectomy specimen. (a) Macroscopic findings. White arrow indicates tumor lesion in the left ureter. (b) Hematoxylin–eosin staining of tumor specimen (200 × magnification). White bar at the bottom right corner indicates 100 μm. (c) Hematoxylin–eosin staining of grade 1 uterine endometrial carcinoma in the original total hysterectomy specimen (200 × magnification). White bar at the bottom right corner indicates 100 μm. (d–f) Positive immunohistochemical stainings for estrogen receptor (d) and PAX8 (e) and negative immunohistochemical staining for GATA3 (f). White bar at the bottom right corner indicates 100 μm (200 × magnification).

**Fig. 5:**
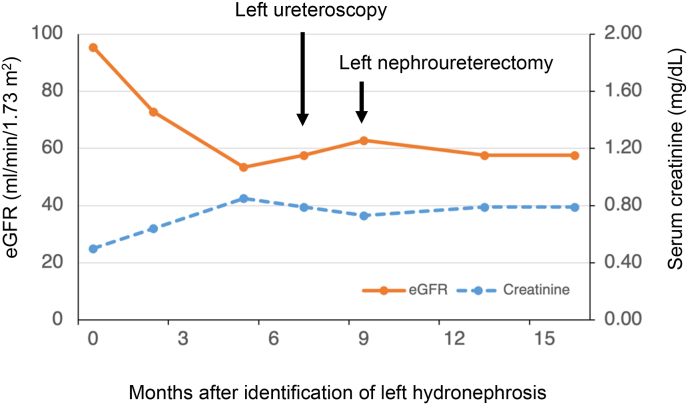
Blood test results for renal function during follow-up. Serum creatinine and eGFR levels after identification of left hydronephrosis.

Given the strong radiologic and pathological suspicion of urothelial carcinoma in the left ureter, we proceeded with left radical nephroureterectomy. Intraoperatively, extensive adhesions involving the peritoneum and pelvic vessels were noted, suggesting local invasion. Unexpectedly, histopathology of the surgical specimen did not reveal any evidence of urothelial carcinoma but showed multiple tubular tumor structures in the ureteral lumen and in the ureteral muscle, histologically resembling the original uterine endometrioid carcinoma in the total hysterectomy specimen (Fig. 4a–c). Immunohistochemical analysis of these tumors revealed positive stainings for estrogen receptor and PAX8 and negative stainings for GATA3, consistent with metastatic uterine endometrioid carcinoma rather than primary UTUC (Fig. 4d–f). The tumor resection margin was negative, and the patient remains disease-free under close surveillance without adjuvant therapy for 6 months. Moreover, the patient's renal function remained stable postoperatively (Fig. 5), consistent with the left kidney being non-functional prior to surgery, as indicated by the 99mTC-MAG3 renography.

## 3 Discussion

In this case, CT and CTU suggested UTUC. Although ureteroscopic biopsy did not yield a definitive pathological diagnosis, malignant cells indicated the presence of urothelial carcinoma. However, nephroureterectomy subsequently revealed metastatic uterine endometrioid carcinoma.

Single-site ureteral metastasis of endometrioid carcinoma is extremely rare. Tsurumaki et al. reported a recurrence in the upper urinary tract 11 years after uterine cancer surgery and suggested implantation during partial ureterectomy with recurrent pelvic lymph node dissection 8 years after hysterectomy as the possible route of metastasis.^7^ In contrast, Salerno et al. reported malignant transformation of endometriosis in the ureter 8 years after hysterectomy for uterine endometrioid cancer.^8^ While malignant transformation from ectopic endometriosis in the ureter might be possible, no evidence of endometriosis was observed in our case. Given the patient's previous pelvic surgery, implantation metastasis was considered to be most likely. Sahl et al. also reported that serous endometrial carcinoma metastasized to the ureter and renal pelvis 3 years after total hysterectomy.^9^ Immunohistochemical analysis of the tumor specimen demonstrated that the tumor was PAX8 positive and GATA3 negative, consistent with the pathological findings of the current case.

CTU has high sensitivity (92 %) and specificity (95 %) for diagnosis of UTUC.^5^^,^^10^ However, CTU has also identified mimics of UTUC.^11^ Benign conditions mistaken for UTUC include ureteral tuberculosis, fibrous polyps, ureteral stones with low CT density, and endometriosis involving the distal ureter.^11^ Metastatic lesions to the ureter are exceedingly rare, with an incidence of approximately 0.37 %.^12^ Karaosmanoglu et al. reported that ureteral metastasis usually involves a relatively short segment with a single lesion, whereas primary UTUC affects a long segment with multifocal lesions.^13^ In this case, the tumor lesion was solitary but relatively large (over 30 mm), consistently suggesting that CTU alone may have limited ability to distinguish ureteral metastasis from primary UTUC.^13^ Therefore, pathological diagnosis using ureteroscopic biopsy is necessary for definitive diagnosis.

MRI can be a useful preoperative diagnostic modality for evaluation of the primary tumor and lymph node metastases in endometrial cancer.^14^ Malignant invasion may be indicated by morphological features such as a spherical or irregular contour, changes in signal intensity on T2-weighted images, central necrosis, or nodal conglomeration on MRI.^15^ Although no characteristic MRI findings specific to ureteral metastasis have been reported and we could not perform MRI in our case because of the patient's claustrophobia, MRI might have distinguished ureteral metastasis from primary ureteral cancer.

Serum CA125 levels can be a useful tumor marker for preoperative diagnosis of endometrial cancer given that elevated CA125 has been reported in patients with primary or recurrent endometrial cancer. A retrospective review of 210 patients with endometrial cancer found that elevated serum CA125 was a strong predictor of extrauterine disease and mortality.^16^ Povolotskaya et al. found that a preoperative CA125 cutoff value of 28 U/mL was a better predictor of overall survival than the conventional value of 35 U/mL.^17^ In our case, the preoperative CA125 level was 7.4 U/mL, which would not have raised suspicion for recurrence even if the cutoff value of CA125 had been set at 28 U/mL.

Finally, if the pathological diagnosis of the biopsy specimen had revealed ureteral metastasis of endometrial carcinoma in advance, partial ureterectomy could have been a treatment option since only a single tumor lesion was observed in the current case. However, since the renal function in this case moderately decreased 2 months after the identification of left hydronephrosis, earlier pathological diagnosis would have been necessary to preserve renal function.

## 4 Conclusion

This case highlights that metastatic relapse of uterine endometrioid carcinoma can mimic ureteral cancer on CTU. When urine cytology and ureteroscopic biopsy are inconclusive, prior malignancies should not be excluded from the differential diagnosis.

## CRediT authorship contribution statement

**Yuji Fujizuka:** Writing – original draft, Visualization, Data curation, Conceptualization. **Shugo Harashima:** Writing – original draft, Visualization, Data curation, Conceptualization. **Yoshiyuki Miyazawa:** Writing – original draft, Visualization, Data curation, Conceptualization. **Seiji Arai:** Writing – review & editing, Writing – original draft, Visualization, Validation, Supervision, Project administration, Data curation, Conceptualization. **Nozomi Nakajima:** Visualization, Data curation. **Mai Onose:** Validation. **Sho Watanuki:** Validation. **Takanori Shimizu:** Validation. **Yoshitaka Sekine:** Validation. **Hidekazu Koike:** Validation. **Hayato Ikota:** Visualization, Supervision. **Kazuhiro Suzuki:** Supervision.

## Informed consent

We obtained informed consent for publication of this report from the patient.

## Approval of the research protocol by an institutional reviewer board

Not applicable.

## Registry and the registration number of the study/trial

Not applicable.

## Conflict of interest

The authors declare no conflict of interest.
